# Information Transfer by Near-Infrared Surface-Plasmon-Polariton Waves on Silver/Silicon Interfaces

**DOI:** 10.1038/s41598-019-48575-6

**Published:** 2019-08-20

**Authors:** Rajan Agrahari, Akhlesh Lakhtakia, Pradip K. Jain

**Affiliations:** 1 Department of Electronics Engineering, Indian Institute of Technology (BHU), Varanasi, 221005 India; 2 NanoMM–Nanoengineered Metamaterials Group, Department of Engineering Science and Mechanics, Pennsylvania State University, University Park, Pennsylvania, 16802 USA; 3 Material Architecture Centre, Indian Institute of Technology (BHU), Varanasi, 221005 India; 4 Department of Electronics and Communications, National Institute of Technology, Patna, 800005 India

**Keywords:** Optics and photonics, Optical materials and structures, Optoelectronic devices and components, Nanophotonics and plasmonics

## Abstract

Electronic interconnections restrict the operating speed of microelectronic chips as semiconductor devices shrink. As surface-plasmon-polariton (SPP) waves are localized, signal delay and crosstalk may be reduced by the use of optical interconnections based on SPP waves. With this motivation, time-domain Maxwell equations were numerically solved to investigate the transport of information by an amplitude-modulated carrier SPP wave guided by a planar silicon/silver interface in the near-infrared spectral regime. The critical-point model was used for the permittivity of silicon and the Drude model for that of silver. The signal can travel long distances without significant loss of fidelity, as quantified by the Pearson and concordance correlation coefficients. The signal is partially reflected and partially transmitted without significant loss of fidelity, when silicon is terminated by air; however, no transmission occurs when silicon is terminated by silver. The fidelity of the transmitted signal in the forward direction rises when both silicon and silver are terminated by air. Thus, signals can possibly be transferred by SPP waves over several tens of micrometers in microelectronic chips.

## Introduction

The prolonged rapid growth of the capabilities of microelectronic circuitry, forecasted about 55 years ago^1^, is beginning to slow down as the silicon-based complementary metal-oxide-semiconductor (CMOS) fabrication technology has matured. The demand for smaller and faster electronic devices appears insatiable, leading researchers to other avenues to enhance the performance of silicon microelectronics.

Devices in a circuit must be interconnected, so faster interconnections will definitely speed up microelectronic chips. Thermal radiation associated with electronic interconnections inhibits much higher data-transfer rates than presently achieved or achievable. Furthermore, electronic interconnections are limited by signal delay and low interconnection density. Optical interconnections inside microelectronic chips can have higher data-transfer rates but the interconnection density is lower than of electronic interconnections because the size of the optical interconnection must exceed the diffraction limit^2,3^.

Optical interconnections exploiting surface-plasmon-polariton (SPP) waves may help greatly enhance data transfer rates in highly integrated optoelectronic circuits on the silicon platform. The strong localization feature of SPP waves can bridge the size mismatch between transistors and optical interconnections^4–6^. Progress on silicon-photonics devices has been impressive for about two decades^7,8^, and the realization of silicon-based electrical sources of SPP waves using CMOS technology^9^ is a significant step towards integrated optoelectronic circuits.

The propagation of an SPP wave is guided by the planar interface of a metal and a dielectric material^10–12^. The plasmonic component of the SPP wave results from the collective oscillation of free electrons (plasma oscillation) on the surface of the metal illuminated by an electromagnetic wave. The polaritonic component of the SPP wave results from the collective oscillation of atomic and molecular dipole moments in the similarly illuminated dielectric material. When the dielectric partnering material is homogeneous, the amplitude of the electric field of the SPP wave is maximum at the interface and decreases with the distance on the both sides of the interface. Therefore, the electromagnetic fields of the SPP waves are highly localized to the interface. Not only is this localization a desirable quality for optical sensing^13^, but it is also attractive for thin optical interconnections in microelectronic chips^5,6^.

The excitation and propagation of SPP waves are typically analyzed after assuming that the electromagnetic fields vary harmonically in time with frequency *f*^10–12^. While this analysis is suitable for optical-sensing applications, it is unwieldy for signals that must be transported by SPP waves in optical interconnections. This is because a signal exists for a finite duration so that frequency-domain analysis of the Maxwell equations must be undertaken for a wide frequency range^14,15^. Direct time-domain analysis^16,17^ using the finite-difference time-domain (FDTD) method^18,19^ is straightforward for theoretical investigation of SPP-wave-based optical communication.

**Figure 1:**
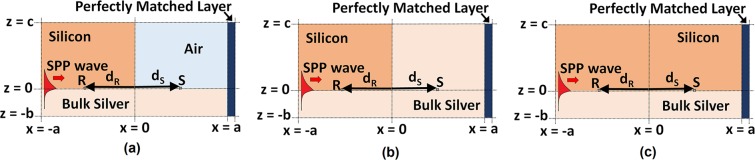
Schematic of the computational domain of the initial-boundary-value problem for information transfer by an amplitude-modulated SPP wave guided by a silver/silicon interface across a wall between silicon and another material. The signal is launched on the plane $x=-\,a$ at time $t=0$ and the wall between silver and either (**a**) air or (**b**) silver is identified as $\{x=0,-\,\infty  < y < \infty ,z > 0\}$. (**c**) Silicon continues beyond the plane $x=0$ in the half space $z > 0$.

With this motivation, we undertook a foundational investigation and solved the Maxwell equations in the time domain to investigate the transport of information by a carrier SPP wave guided by a planar silicon/silver interface. The critical-point model was used for the frequency-domain relative permittivity of silicon^20,21^ and the Drude model for that of silver^22,23^, and the frequency-domain data was converted to the time domain using the inverse Fourier transform^24^. The carrier wavelength ${\lambda }_{{\rm{c}}}=1200\,{\rm{nm}}$ was chosen to lie in the near-infrared spectral regime. In the FDTD simulation, a carrier SPP wave with amplitude modulated by a signal pulse was launched at a specific location on the silicon/silver interface and allowed to propagate a certain distance before it encountered an upright wall between silicon and another material, which could be either air or silver, as shown in Fig. 1(a,b). The transmission of the signal beyond this wall was simulated in order to determine the fidelity of information transfer beyond the wall, as assessed using the Pearson^25^ and the concordance^26^ correlation coefficients.

The remainder of this paper is organized as follows. First, we briefly present the geometry of the problem and the time-domain constitutive relations of the various materials involved, and then we present numerical results to determine the fidelity of information transfer.

## Problem Geometry and Constitutive Relations

The geometry of the chosen problem is shown in Fig. 1. Relevant to the FDTD simulation, the spatial domain is $${\mathscr{R}}:\{|x|\le a,-\,{\rm{\infty }} < y < {\rm{\infty }},-\,b\le z\le c\}$$ and the temporal domain is $${\mathscr{T}}:\{t\ge 0\}$$. Thus, $$ {\mathcal R} \times {\mathscr{T}}$$ is the computational domain. The spatial domain $$ {\mathcal R} $$ is partitioned into four subdomains identified as $${ {\mathcal R} }_{A}:\{-a\le x < 0,$$$$-\,\infty  < y < \infty ,0 < z\le c\}$$, $${ {\mathcal R} }_{B}:\{-a\le x < 0,-\,\infty  < y < \infty ,-\,b\le z < 0\}$$, $${ {\mathcal R} }_{C}:\{0 < x\le a,-\,{\rm{\infty }} < y < {\rm{\infty }},$$$$0\le z\le c\},$$ and $${ {\mathcal R} }_{D}:\{0\le x < a,-\,\infty  < y < \infty ,-\,b\le z < 0\}$$. The subdomain $${ {\mathcal R} }_{A}$$ is occupied by a homogeneous dielectric material (silicon) and the subdomains $${ {\mathcal R} }_{B}$$ and $${ {\mathcal R} }_{D}$$ by a homogeneous metal (bulk silver). Information transfer by the carrier SPP wave was determined for three different materials occupying the subdomain $${ {\mathcal R} }_{C}$$:

(i) air (Fig. 1a),
(ii) silver (Fig. 1b), and
(iii) silicon (Fig. 1c).

In order to analyze the fidelity of information transfer, we identified a point labeled R $({x}_{{\rm{R}}}=-\,{d}_{{\rm{R}}},{z}_{{\rm{R}}}={0}^{+})$ in ${ {\mathcal R} }_{A}$ as the point of transmission and a point labeled S $({x}_{{\rm{S}}}={d}_{{\rm{S}}},{z}_{{\rm{S}}}={0}^{+})$ in ${ {\mathcal R} }_{C}$ as the point of reception, as shown in Fig. 1.

The frequency-domain relative permittivity of silicon is described by the critical-point model as^20,21^

1 $${\tilde{\varepsilon }}_{{\rm{Si}}}(\omega )=1+{\zeta }_{1}[\frac{{{\rm{\Omega }}}_{1}^{2}-i{\Upsilon }_{1}\omega }{{{\rm{\Omega }}}_{1}^{2}-2i{{\rm{\Gamma }}}_{1}\omega -{\omega }^{2}}]+{\zeta }_{2}[\frac{{{\rm{\Omega }}}_{2}^{2}-i{\Upsilon }_{2}\omega }{{{\rm{\Omega }}}_{2}^{2}-2i{{\rm{\Gamma }}}_{2}\omega -{\omega }^{2}}],$$

where $i=\sqrt{-\,1}$ is the imaginary unit; $\omega =2\pi f$ is the angular frequency; and the constants ${\zeta }_{1}=8.93$, ${\zeta }_{2}=1.855$, ${{\rm{\Omega }}}_{1}=6.4465\times {10}^{15}\,{\rm{rad}}\,{{\rm{s}}}^{-1}$, ${{\rm{\Omega }}}_{2}=5.1271\times {10}^{15}\,{\rm{rad}}\,{{\rm{s}}}^{-1}$, ${\Upsilon }_{1}=1.6399\times {10}^{14}\,{\rm{rad}}\,{{\rm{s}}}^{-1}$, ${\Upsilon }_{2}=5.0479\times$ ${10}^{15}\,{\rm{rad}}\,{{\rm{s}}}^{-1}$, ${{\rm{\Gamma }}}_{1}=8.0111\times {10}^{14}\,{\rm{rad}}\,{{\rm{s}}}^{-1}$, and ${{\rm{\Gamma }}}_{2}=2.3185\times {10}^{14}\,{\rm{rad}}\,{{\rm{s}}}^{-1}$ were chosen^21^ to best fit experimental data^27^. The inverse Fourier transform^24^ yields the time-domain relative permittivity of silicon as

2 $$\begin{array}{rcl}{\varepsilon }_{{\rm{Si}}}(t) & = & \delta (t)+\mathop{\sum }\limits_{p=1}^{2}\{{\zeta }_{p}\,\exp (\,-{{\rm{\Gamma }}}_{p}t)[\tfrac{{{\rm{\Omega }}}_{p}^{2}-{\Upsilon }_{p}{{\rm{\Gamma }}}_{p}}{{\alpha }_{p}}\,\sin ({\alpha }_{p}t)\\  &  & +\,{\Upsilon }_{p}\,\cos ({\alpha }_{p}t)]\}{\mathscr{U}}(t),\end{array}$$

where *δ*(*t*) is the Dirac delta, ${\mathscr{U}}(t)$ is the unit step function, and ${\alpha }_{p}=+\,\sqrt{{{\rm{\Omega }}}_{p}^{2}-{{\rm{\Gamma }}}_{p}^{2}}$, $p\in \{1,2\}$.

The frequency-domain relative permittivity of bulk silver is described by the Drude model as^22,23^

3 $${\tilde{\varepsilon }}_{{\rm{Ag}}}(\omega )=1-\{\frac{{\omega }_{{\rm{Ag}}}^{2}}{\omega (\omega +i/{\tau }_{{\rm{Ag}}})}\},$$

where ${\omega }_{{\rm{Ag}}}=1.352\times {10}^{16}\,{\rm{rad}}\,{{\rm{s}}}^{-1}$ and ${\tau }_{{\rm{Ag}}}=1.7\times {10}^{-14}\,{\rm{s}}$, respectively, are the plasma angular frequency and the relaxation time. The inverse Fourier transform yields the time-domain relative permittivity of bulk silver as

4 $${\varepsilon }_{{\rm{Ag}}}(t)=\delta (t)+{\omega }_{{\rm{Ag}}}^{2}{\tau }_{{\rm{Ag}}}[1-\exp (\,-\frac{t}{{\tau }_{{\rm{Ag}}}})]{\mathscr{U}}(t\mathrm{).}$$

In our FDTD simulations, $\partial /\partial y\equiv 0$ is set in the time-domain Maxwell curl equations. Furthermore, the amplitude of the electric field of the carrier SPP wave on the plane $x=-\,a$ is modulated by the pulse function

5 $$g(t)={\omega }_{{\rm{c}}}t\,\exp (\,-\,{\omega }_{{\rm{c}}}t),$$

where ${\omega }_{c}=2\pi {c}_{0}/{\lambda }_{c}$ is the angular frequency of the carrier SPP wave and ${c}_{0}=3\times {10}^{8}\,{\rm{m}}\,{{\rm{s}}}^{-1}$ is the speed of light in free space. Thus, the electric field ${\bf{E}}(x,z,t)$ on the plane $x=-\,a$ for all $t\in T$ is specified as^16^

6 $${\bf{E}}(\,-\,a,z,t)=\{\begin{array}{ll}g(t)\,{\rm{Re}}[\tfrac{-{\alpha }_{{{\rm{c}}}_{{\rm{Si}}}}{\hat{{\bf{u}}}}_{x}+{q}_{{\rm{c}}}{\hat{{\bf{u}}}}_{z}}{{k}_{{\rm{c}}}{\tilde{\varepsilon }}_{{\rm{Si}}}({\omega }_{{\rm{c}}})}\exp (i{\alpha }_{{{\rm{c}}}_{{\rm{Si}}}}z)\,\exp (-i{\omega }_{{\rm{c}}}t)], & z > \mathrm{0,}\\ g(t)\,{\rm{Re}}[\tfrac{{\alpha }_{{{\rm{c}}}_{{\rm{Ag}}}}{\hat{{\bf{u}}}}_{x}+{q}_{{\rm{c}}}{\hat{{\bf{u}}}}_{z}}{{k}_{{\rm{c}}}{\tilde{\varepsilon }}_{{\rm{Ag}}}({\omega }_{{\rm{c}}})}\exp (-i{\alpha }_{{{\rm{c}}}_{{\rm{Ag}}}}z)\,\exp (-i{\omega }_{{\rm{c}}}t)], & z < \mathrm{0,}\end{array}$$

where ${\hat{{\bf{u}}}}_{x}$ and ${\hat{{\bf{u}}}}_{z}$ are Cartesian unit vectors along the *x* and *z* axes, respectively. The free-space wavenumber of the carrier SPP wave in free space is denoted by ${k}_{{\rm{c}}}={\omega }_{{\rm{c}}}/{c}_{0}$;

7 $${q}_{{\rm{c}}}={k}_{{\rm{c}}}\sqrt{\frac{{\tilde{\varepsilon }}_{{\rm{Si}}}({\omega }_{{\rm{c}}})\,{\tilde{\varepsilon }}_{{\rm{Ag}}}({\omega }_{{\rm{c}}})}{{\tilde{\varepsilon }}_{{\rm{Si}}}({\omega }_{{\rm{c}}})+{\tilde{\varepsilon }}_{{\rm{Ag}}}({\omega }_{{\rm{c}}})}}$$

is the complex wavenumber describing the propagation and attenuation of the carrier SPP wave along the silicon/silver interface^10–12^; and the complex wavenumbers

8 $${\alpha }_{{{\rm{c}}}_{{\rm{Si}}}}=\sqrt{{k}_{{\rm{c}}}^{2}{\tilde{\varepsilon }}_{{\rm{Si}}}({\omega }_{{\rm{c}}})-{q}_{{\rm{c}}}^{2}}$$

and

9 $${\alpha }_{{{\rm{c}}}_{{\rm{Ag}}}}=\sqrt{{k}_{{\rm{c}}}^{2}{\tilde{\varepsilon }}_{{\rm{Ag}}}({\omega }_{{\rm{c}}})-{q}_{{\rm{c}}}^{2}}$$

describe field variation in the *z* direction. The conditions ${\rm{Re}}({q}_{{\rm{c}}}){\rm{Im}}({q}_{{\rm{c}}}) > 0$, ${\rm{Im}}({\alpha }_{{{\rm{c}}}_{{\rm{Si}}}}) > 0$, and ${\rm{Im}}({\alpha }_{{{\rm{c}}}_{{\rm{Ag}}}}) > 0$ apply. Corresponding expressions for the magnetic field ${\bf{H}}(x,z,t)$ on the plane $x=-\,a$ for all $t\in T$ are available elsewhere^16^.

**Figure 2:**
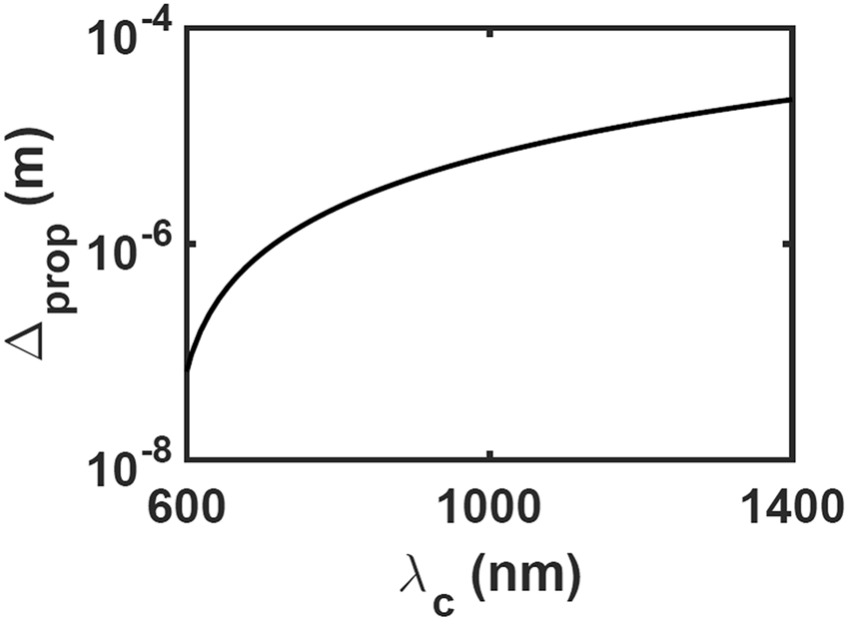
Variation of the propagation distance Δ_prop_ of the carrier SPP wave with the free-space wavelength *λ*_c_ when the partnering materials are silicon and silver.

The dimension *a* of the computational domain and the carrier wavelength *λ*_c_ have to be chosen keeping the carrier SPP wave’s propagation distance ${{\rm{\Delta }}}_{{\rm{prop}}}=1/{\rm{Im}}({q}_{{\rm{c}}})$ along the *x* axis in mind. The variation of Δ_prop_ with *λ*_c_ is shown in Fig. 2. Whereas Δ_prop_ is very small in the visible spectral regime, it is considerably higher in the near-infrared spectral regime. Moreover, *λ*_c_ must be sufficiently removed from the telecommunication regime $[1260,1625]\,{\rm{nm}}$ to prevent interference, if the microchip is to be used in a telecommunication network. Therefore we chose ${\lambda }_{{\rm{c}}}=1200\,{\rm{nm}}$ for all numerical results presented here, although our qualitative conclusions apply for higher values of *λ*_c_ as well. Since Δ_prop_ = 13.21 *μ*m for ${\lambda }_{{\rm{c}}}=1200\,{\rm{nm}}$, we fixed $a=5000\,{\rm{nm}}$.

Furthermore, the dimensions *b* and *c* must be much larger than the depth of penetration of the carrier SPP wave in silver and silicon, respectively^16^, so that reflections from the planes $z=-\,b$ and $z=c$ into ${\mathcal R}$ are minuscule when the FDTD method is implemented. We set $b=203\,{\rm{nm}}$ and $c=949\,{\rm{nm}}$ after examining the values of ${\alpha }_{{{\rm{c}}}_{{\rm{Ag}}}}$ and ${\alpha }_{{{\rm{c}}}_{{\rm{Si}}}}$ for ${\lambda }_{{\rm{c}}}=1200\,{\rm{nm}}$.

In order to implement the FDTD method, ${\mathcal R}$ is discretized into Δ*x* × Δ*z* rectangular cells, ${\mathscr{T}}$ is discretized into linear cells of duration Δ*t*, and derivatives are approximated using the central difference formula^18^. The physical domain ${\mathcal R}$ is encapsulated by a perfectly match layer to the right of the plane $x=a$ in order to prevent reflection into ${\mathcal R}$^16^. Details of the perfectly matched layer and the FDTD updating equations are available elsewhere^16^. We set ${\rm{\Delta }}x=25\,{\rm{nm}}$, ${\rm{\Delta }}z=5.76\,{\rm{n}}{\rm{m}},$ and ${\rm{\Delta }}t=0.017\,{\rm{fs}}$ so as to satisfy the Courant–Friedrichs–Lewy criterion^19^.

## Numerical Results and Discussion

The amplitude-modulated carrier SPP wave is launched in the computational domain ${\mathcal R} \times {\mathscr{T}}$ from the plane $x=-\,a$ at $t=0$, as depicted in Fig. 1. In order to determine the transmission of the information, we evaluated the temporal variation of the instantaneous Poynting vector

10 $${\bf{P}}(x,z,t)={\bf{E}}(x,z,t)\times {\bf{H}}(x,z,t)$$

at the transmission point R and the reception point S.

**Figure 3:**
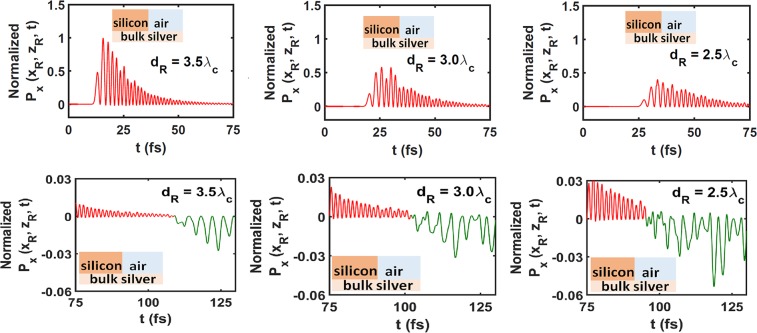
Temporal profile of normalized ${P}_{x}({x}_{{\rm{R}}},{z}_{{\rm{R}}},t)$ when ${ {\mathcal R} }_{C}$ is occupied by air, ${ {\mathcal R} }_{D}$ is occupied by silver, and ${\lambda }_{{\rm{c}}}=1200\,{\rm{nm}}$. Left column: ${d}_{{\rm{R}}}=3.5{\lambda }_{{\rm{c}}}$, middle column: ${d}_{{\rm{R}}}=3.0{\lambda }_{{\rm{c}}}$, and right column: ${d}_{{\rm{R}}}=2.5{\lambda }_{{\rm{c}}}$. The top row shows the transmitted signal (red curve) at point R and the bottom row shows the tail of the transmitted signal (red curve) followed by the reflected signal (green curve) at point R. Multiply by 6.8 × 10^−6^ W m^−2^ to obtain unnormalized ${P}_{x}({x}_{{\rm{R}}},{z}_{{\rm{R}}},t)$.

Figure 3 presents the temporal profile of the axial component ${P}_{x}({x}_{{\rm{R}}},{z}_{{\rm{R}}},t)={\hat{{\bf{u}}}}_{x}\cdot {\bf{P}}({x}_{{\rm{R}}},{z}_{{\rm{R}}},t)$ of the Poynting vector at the transmission point for three different values of *d*_R_ when the subdomain ${ {\mathcal R} }_{C}$ is occupied by air. All plots of the components of the instantaneous Poynting vector in this paper are normalized with respect to magnitude 6.8 × 10^−6^ W m^−2^. The top row of the Fig. 3 shows ${P}_{x}({x}_{{\rm{R}}},{z}_{{\rm{R}}},t)$ for $t\in [0,75]\,{\rm{fs}}$ and the bottom row for $t\in [75,130]\,{\rm{fs}}$. Thus, the top row shows the transmitted signal and the bottom row shows the tail of the transmitted signal followed by the reflected signal at point R.

The transmitted signal exists for $t\in [11.2,51.31]\,{\rm{fs}}$, $t\in [17.95,63.45]\,{\rm{fs}}$, and $t\in [25.4,73.17]\,{\rm{fs}}$ when ${d}_{{\rm{R}}}=3.5{\lambda }_{{\rm{c}}}$, ${d}_{{\rm{R}}}=3.0{\lambda }_{{\rm{c}}}$, and ${d}_{{\rm{R}}}=2.5{\lambda }_{{\rm{c}}}$, respectively (see Fig. 3, top row). If we quantify the signal duration as the time interval for which ${P}_{x}({x}_{{\rm{R}}},{z}_{{\rm{R}}},t)$ exceeds 50% of its peak value, the signal duration is 12.26 fs for ${d}_{{\rm{R}}}=3.5{\lambda }_{{\rm{c}}}$, 13.22 fs for ${d}_{{\rm{R}}}=3.0{\lambda }_{{\rm{c}}}$, and 15.57 fs for ${d}_{{\rm{R}}}=2.5{\lambda }_{{\rm{c}}}$. The increasing duration of the transmitted signal as the point R is chosen closer to the plane $x=0$ indicates that the pulse broadens as it propagates guided by the silicon/silver interface. This observation is consistent with different spectral components of the transmitted signal having different phase speeds because of the $\omega$-dependence of ${\tilde{\varepsilon }}_{{\rm{Si}}}$ and ${\tilde{\varepsilon }}_{{\rm{Ag}}}$. Furthermore, the peak intensity of the pulse at R decreases in Fig. 3 (top row), if that point is chosen closer to the plane $x=0$. This observation is consistent with attenuation of the carrier SPP wave because silver (${\tilde{\varepsilon }}_{{\rm{Ag}}}=-\,72.9784+2.7704i$) is highly dissipative at ${\lambda }_{{\rm{c}}}=1200\,{\rm{nm}}$ although dissipation in silicon (${\tilde{\varepsilon }}_{{\rm{Si}}}=12.5224+0.0043i$) is negligbly small.

Figure 3 (bottom row) shows that the reflected signal begins to appear at $t=109\,{\rm{fs}}$, $t=103\,{\rm{fs}}$, and $t=95.35\,{\rm{fs}}$ for ${d}_{{\rm{R}}}=3.5{\lambda }_{{\rm{c}}}$, ${d}_{{\rm{R}}}=3.0{\lambda }_{{\rm{c}}}$, and ${d}_{{\rm{R}}}=2.5{\lambda }_{{\rm{c}}}$, respectively. Most of the transmitted signal is reflected by the plane $x=0$ because the subdomain ${ {\mathcal R} }_{C}$ is occupied by air. Furthermore, the peak intensity of the reflected signal at R increases, if that point is chosen closer to the plane $x=0$.

**Figure 4:**
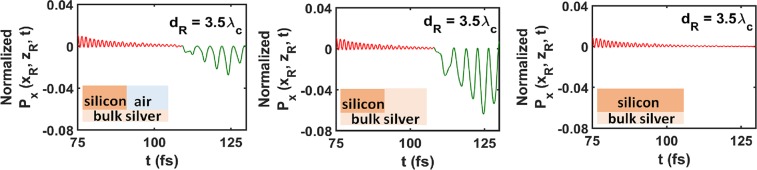
Temporal profile of normalized ${P}_{x}({x}_{{\rm{R}}},{z}_{{\rm{R}}},t)$ when ${\lambda }_{{\rm{c}}}=1200\,{\rm{nm}}$; ${d}_{{\rm{R}}}=3.5{\lambda }_{{\rm{c}}}$; ${ {\mathcal R} }_{C}$ is occupied by (left) air, (middle) silver, and (right) silicon; and ${ {\mathcal R} }_{D}$ is occupied by silver. Each profile is of the tail of the transmitted signal (red curve) followed by the reflected signal (green curve) at point R. Multiply by 6.8 × 10^−6^ W m^−2^ to obtain unnormalized ${P}_{x}({x}_{{\rm{R}}},{z}_{{\rm{R}}},t)$.

The dependence of the reflected signal on the material occupying the subdomain ${ {\mathcal R} }_{C}$ can be gleaned from the plots of ${P}_{x}({x}_{{\rm{R}}},{z}_{{\rm{R}}},t)$ vs. $t\in [75,130]\,{\rm{fs}}$ for ${d}_{{\rm{R}}}=3.5{\lambda }_{{\rm{c}}}$ in Fig. 4. Each plot contains the tail of the transmitted signal followed by the reflected signal at point R. When the materials in the subdomains ${ {\mathcal R} }_{B}$ and ${ {\mathcal R} }_{C}$ are different (silicon and air, or silicon and silver), their impedance mismatch is responsible for most of the transmitted signal being reflected by the discontinuity at $\{x=0,z > 0\}$. Figure 4 (left) and (middle) show reflection at point R when ${ {\mathcal R} }_{C}$ is occupied by air and silver, respectively. Since the metal does not allow propagation of an electromagnetic wave inside it beyond a short distance, the peak intensity of the reflected signal is found higher when ${ {\mathcal R} }_{C}$ is occupied by silver than when it is occupied by air. Furthermore, the intensity of the reflected signal in Fig. 4 (right) is zero, since there is no discontinuity at the plane $x=0$ when ${ {\mathcal R} }_{C}$ is occupied by silicon.

**Figure 5:**
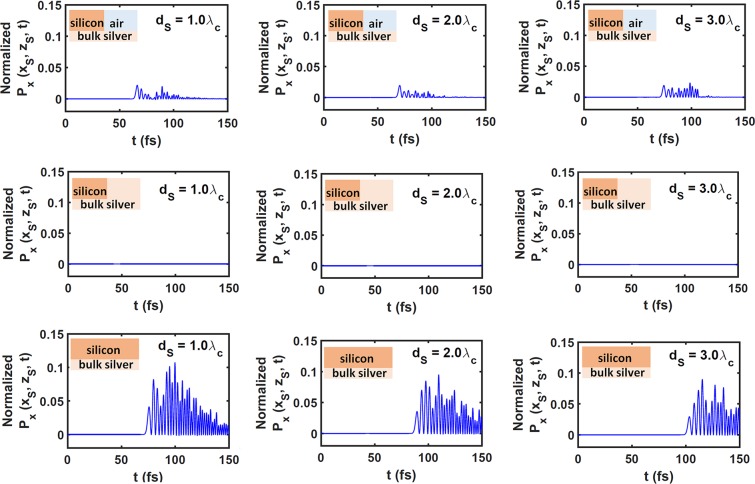
Temporal profile of normalized ${P}_{x}({x}_{{\rm{S}}},{z}_{{\rm{S}}},t)$ when ${\lambda }_{{\rm{c}}}=1200\,{\rm{nm}}$ and ${ {\mathcal R} }_{D}$ is occupied by silver. Top row: ${ {\mathcal R} }_{C}$ is occupied by air, middle row: ${ {\mathcal R} }_{C}$ is occupied by silver, and bottom row: ${ {\mathcal R} }_{C}$ is occupied by silicon. Left column: ${d}_{{\rm{S}}}/{\lambda }_{{\rm{c}}}=1$, middle column: ${d}_{{\rm{S}}}/{\lambda }_{{\rm{c}}}=2$, and right column: ${d}_{{\rm{S}}}/{\lambda }_{{\rm{c}}}=3$. Each profile is of the received signal at point S. Multiply by 6.8 × 10^−6^ W m^−2^ to obtain unnormalized ${P}_{x}({x}_{{\rm{S}}},{z}_{{\rm{S}}},t)$.

The signal received at the reception point S also depends on the material in the subdomain ${ {\mathcal R} }_{C}$. Figure 5 provides temporal profiles of ${P}_{x}({x}_{{\rm{S}}},{z}_{{\rm{S}}},t)$ for ${d}_{{\rm{S}}}/{\lambda }_{{\rm{c}}}\in \{1.0,2.0,3.0\}$ and ${\lambda }_{{\rm{c}}}=1200\,{\rm{nm}}$, when ${ {\mathcal R} }_{C}$ is occupied by air, silver, and silicon. A comparison of the left panel of Fig. 4 with the top row of Fig. 5 shows that the energy of the received signal is weaker than that of the reflected signal, when ${ {\mathcal R} }_{C}$ is occupied by air. Thus, reflection by the silicon/air interface $\{x=0,z > 0\}$ is highly significant. Furthermore, since the skin depth^28^ of silver is minuscule, the middle row of Fig. 5 shows the intensity of the received signal is infinitesimal when ${ {\mathcal R} }_{C}$ is occupied by silver. Finally, the bottom row of Fig. 5 presents the received signal at S when ${ {\mathcal R} }_{C}$ is occupied by silicon. In this case, the intensity of the received signal is stronger than when ${ {\mathcal R} }_{C}$ is occupied by air. This is because of zero reflection of the transmitted signal by the plane $x=0$, as shown in right panel of Fig. 4.

The signal received at S is a distorted version of the transmitted signal at R. The top and bottom rows of Fig. 5 show that the shape of the received signal is approximately the same as that of transmitted signal but the duration is not the same. The duration of the received signal is invariant with respect to *d*_S_ when ${ {\mathcal R} }_{C}$ is occupied by air, which is explained by the fact that air is a non-dispersive material (in the present context). However, the duration of the received signal increases with *d*_S_ when ${ {\mathcal R} }_{C}$ is occupied by the silicon, which is consistent with the signal broadening with increasing *d*_R_ observed in the top row of Fig. 3.

The signal received at point S in Fig. 5 (top row) is definitely dependent on *d*_S_, because silicon is replaced by air across the plane $x=0$. The signal received at point S in Fig. 5 (middle row) is definitely independent *d*_S_, because the chosen values of *d*_S_ are significantly larger than the skin depth in silver. The signal received at point S in Fig. 5 (bottom row) is definitely dependent on *d*_S_, because this signal is simply the transmitted signal in the absence of a material discontinuity across the plane $x=0$. As the transmitted signal propagates farther, it gets distorted more because different spectral components decay at different rates due to both silver and silicon being dispersive.

The first three rows of data in Table 1 provide a comparison of the energy ${{\rm{E}}}_{{\rm{f}}}(x)$ of the forward signal and the energy ${{\rm{E}}}_{{\rm{b}}}(x)$ of the backward (i.e., reflected) signal passing through different planes $x={\rm{const}}.$ when ${ {\mathcal R} }_{C}$ is occupied by air, silver, and silicon and ${ {\mathcal R} }_{D}$ by silver, as depicted in Fig. 1a–c. The net signal energy ${\rm{\Delta }}{\rm{E}}(x)={{\rm{E}}}_{{\rm{f}}}(x)-{{\rm{E}}}_{{\rm{b}}}(x)$ at the plane $x=-\,3.5{\lambda }_{{\rm{c}}}$ exceeds the net signal energy at the plane $x=-\,2.5{\lambda }_{{\rm{c}}}$, regardless of the material occupying ${ {\mathcal R} }_{C}$. This is because E_b_ also diminishes with the propagation distance (in the backward direction).

**Table 1:** Normalized energies of the forward signal (E_f_) and backward signal (E_b_) passing across the plane *x* = const.

${{\boldsymbol{ {\mathcal R} }}}_{{\bf{C}}}$	${{\boldsymbol{ {\mathcal R} }}}_{{\bf{D}}}$	Normalized energy									
		*x* = −3.5*λ*_c_		*x* = −2.5*λ*_c_		*x* = 0^+^		*x* = 1.0*λ*_c_		*x* = 3.0*λ*_c_	
		E_f_	E_b_	E_f_	E_b_	E_f_	E_b_	E_f_	E_b_	E_f_	E_b_
air	silver	1.0	0.1285	0.81	0.1951	0.3102	0.0	0.3058	0.0	0.3033	0.0
silver	silver	1.0	0.2209	0.81	0.3749	0.0006	0.0	0.0	0.0	0.0	0.0
silicon	silver	1.0	0.0	0.81	0.0	0.5883	0.0	0.5304	0.0	0.4044	0.0
air	air	1.0	0.1495	0.81	0.2267	0.2426	0.0	0.2408	0.0	0.2395	0.0
silicon	silicon	1.0	0.0077	0.81	0.0180	0.5117	0.0	0.4961	0.0	0.4270	0.0

As a fraction of ${{\rm{E}}}_{{\rm{f}}}(\,-\,3.5{\lambda }_{{\rm{c}}})$, the signal energy decreases as the amplitude-modulated carrier SPP wave propagates forward. In the absence of a discontinuity across the plane $x=0$, nearly 40% of ${{\rm{E}}}_{{\rm{f}}}(\,-\,3.5{\lambda }_{{\rm{c}}})$ reaches the plane $x=3{\lambda }_{{\rm{c}}}$, i.e., a distance of 9 μm or about 650 transistors of linear size 14 nm laid end to end. That number of transistors will increase in a few years. Even when ${ {\mathcal R} }_{C}$ is occupied by air, about 30% of the forward signal energy at $x=-\,3.5{\lambda }_{{\rm{c}}}$ reaches the plane $x=3{\lambda }_{{\rm{c}}}$. Of course, reflection is maximum when ${ {\mathcal R} }_{C}$ is occupied by silver.

Suppose in Fig. 1, if silver is abruptly terminated at $x=0$ and both subdomains ${ {\mathcal R} }_{C}$ and ${ {\mathcal R} }_{D}$ are occupied by the same material. When this material is silver (Fig. 1b), practically no energy passes forward through the plane $x={0}^{+}$, as is clear from the middle row of Fig. 5 and the second row of data in Table 1. When the material occupying ${ {\mathcal R} }_{C}\cup { {\mathcal R} }_{D}$ is air, the fourth row of data in Table 1 indicates that energy does pass forward through the plane $x={0}^{+}$ and decays very slowly as *x* increases. When the material occupying ${ {\mathcal R} }_{C}\cup { {\mathcal R} }_{D}$ is silicon, the fifth row of data in Table 1 indicates that even more energy passes forward through the plane $x={0}^{+}$ but decays more rapidly as $x$ increases.

The plane $x={0}^{+}$ can be considered to be the virtual source of energy that passes into ${ {\mathcal R} }_{C}\cup { {\mathcal R} }_{D}$. This source is of limited extent along the *z* axis because of the localization feature of SPP waves. As such, the Huygens’ principle indicates that the energy would not pass only in the forward direction but also in other directions, as we verified from our simulations. Therefore, it will decay in all directions. When ${ {\mathcal R} }_{C}\cup { {\mathcal R} }_{D}$ is occupied by silicon rather than air, the decay rate is higher because silicon does have some dissipation because ${\rm{Im}}({\tilde{\varepsilon }}_{{\rm{Si}}}) > 0$ while air is assumed to be non-dissipative.

Reverting to the three situations depicted in Fig. 1a–c, we determined the Pearson^25^ (${\rho }_{{P}_{{\rm{RS}}}}$) and the concordance^26^ (${\rho }_{{C}_{{\rm{RS}}}}$) correlation coefficients, between the transmitted and received signals in order to quantify the similarity of the signal received at point S to the signal transmitted at point R.

The Pearson correlation coefficient ${\rho }_{{P}_{{\rm{RS}}}}\in [\,-\,1,1]$ compares the shapes and durations of two signals. This coefficient is defined as

11 $${\rho }_{{P}_{{\rm{RS}}}}=\frac{{\sum }_{\ell =1}^{N}\,[\{{P}_{x}({x}_{{\rm{R}}},{z}_{{\rm{R}}},{t}_{\ell })-{\mu }_{{\rm{R}}}\}\times \{{P}_{x}({x}_{{\rm{S}}},{z}_{{\rm{S}}},{t}_{\ell }-\bar{t})-{\mu }_{{\rm{S}}}\}]}{N{\sigma }_{{\rm{R}}}{\sigma }_{{\rm{S}}}},$$

where

12 $${\mu }_{{\rm{R}}}=\frac{1}{N}\,\mathop{\sum }\limits_{\ell =1}^{N}\,{P}_{x}({x}_{{\rm{R}}},{z}_{{\rm{R}}},{t}_{\ell }),$$

13 $${\mu }_{{\rm{S}}}=\frac{1}{N}\,\mathop{\sum }\limits_{\ell =1}^{N}\,{P}_{x}({x}_{{\rm{S}}},{z}_{{\rm{S}}},{t}_{\ell }),$$

14 $${\sigma }_{{\rm{R}}}=\sqrt{\frac{1}{N}\,\mathop{\sum }\limits_{\ell =1}^{N}\,{[{P}_{x}({x}_{{\rm{R}}},{z}_{{\rm{R}}},{t}_{\ell })-{\mu }_{{\rm{R}}}]}^{2}},$$

15 $${\sigma }_{{\rm{S}}}=\sqrt{\frac{1}{N}\,\mathop{\sum }\limits_{\ell =1}^{N}\,{[{P}_{x}({x}_{{\rm{S}}},{z}_{{\rm{S}}},{t}_{\ell })-{\mu }_{{\rm{S}}}]}^{2}},$$

and

16 $$\bar{t}=\frac{1}{{c}_{0}}\sqrt{{({x}_{{\rm{S}}}-{x}_{{\rm{R}}})}^{2}+{({z}_{{\rm{S}}}-{z}_{{\rm{R}}})}^{2}}.$$

A higher value of $|{\rho }_{{P}_{{\rm{RS}}}}|$ indicates stronger correlation or anticorrelation, as indicated by the sign of ${\rho }_{{P}_{{\rm{RS}}}}$. If the received signal is independent of the transmitted signal, then ${\rho }_{{P}_{{\rm{RS}}}}=0$.

The concordance correlation coefficient compares the shapes of two signals and is defined as^26^

17 $${\rho }_{{C}_{{\rm{RS}}}}={\rho }_{{P}_{{\rm{RS}}}}{C}_{b},$$

where the bias factor

18 $${C}_{b}=\frac{2}{(\frac{{\sigma }_{{\rm{R}}}}{{\sigma }_{{\rm{S}}}})+(\frac{{\sigma }_{{\rm{S}}}}{{\sigma }_{{\rm{R}}}})+\frac{{({\mu }_{{\rm{R}}}-{\mu }_{{\rm{S}}})}^{2}}{{\sigma }_{{\rm{R}}}{\sigma }_{{\rm{S}}}}}.$$

Since *C*_*b*_ is $[0,1]$ and ${\rho }_{{P}_{{\rm{RS}}}}\in [\,-\,1,\mathrm{1]}$, it follows that ${\rho }_{{C}_{{\rm{RS}}}}\in [\,-\,1,1]$, $0\le {\rho }_{{C}_{{\rm{RS}}}}{\rho }_{{P}_{{\rm{RS}}}}\le 1$, and $|{\rho }_{{C}_{{\rm{RS}}}}|\le |{\rho }_{{P}_{{\rm{RS}}}}|$. If ${\rho }_{{C}_{{\rm{RS}}}}=0$, then the transmitted signal and the received signal have completely different shapes; if ${\rho }_{{C}_{{\rm{RS}}}} < 0$, the two signals are negatively correlated.

Table 2 provides values of ${\rho }_{{P}_{{\rm{RS}}}}$ and ${\rho }_{{C}_{{\rm{RS}}}}$ when ${ {\mathcal R} }_{C}$ is occupied by air, silver, and silicon and ${ {\mathcal R} }_{D}$ by silver for ${d}_{{\rm{S}}}/{\lambda }_{{\rm{c}}}\in \{1.0,2.0,3.0\}$ and ${d}_{{\rm{R}}}=3.5{\lambda }_{{\rm{c}}}$. Since the shape of the received signal is approximately same as that of the transmitted signal when ${ {\mathcal R} }_{C}$ is occupied by either air or silicon, as indicated by the top and bottom rows of Fig. 5, ${\rho }_{{P}_{{\rm{RS}}}} > 0.5$ and ${\rho }_{{C}_{{\rm{RS}}}} > 0.5$ over the chosen range of *d*_S_, thereby confirming that information can indeed be transferred by the carrier SPP wave. Furthermore, ${\rho }_{{C}_{{\rm{RS}}}}\approx 0.5$ means that the received signal is moderately in agreement with the transmitted signal and thus the fidelity of the shape of the received signal is moderate too. Since the duration of the received signal does not equal that of the transmitted signal, as indicated by the top and bottom rows of Fig. 5, we get the moderate value of ${\rho }_{{P}_{{\rm{RS}}}}\approx 0.5$. When both ${ {\mathcal R} }_{C}$ and ${ {\mathcal R} }_{D}$ are occupied by silver, the signal does not travel for an appreciable distance beyond the plane $x={0}^{+}$, as is clear from the middle row of Fig. 5, and both correlation coefficients become meaningless.

**Table 2:** ${\rho }_{{P}_{{\rm{RS}}}}$ and ${\rho }_{{C}_{{\rm{RS}}}}$ for ${d}_{{\rm{S}}}/{\lambda }_{{\rm{c}}}\in \{1.0,2.0,3.0\}$ and ${d}_{{\rm{R}}}=3.5{\lambda }_{{\rm{c}}}$.

*d*_S_/*λ*_c_	${{\boldsymbol{ {\mathcal R} }}}_{{\boldsymbol{C}}}$: air ${{\boldsymbol{ {\mathcal R} }}}_{{\boldsymbol{D}}}$: silver		${{\boldsymbol{ {\mathcal R} }}}_{{\boldsymbol{C}}}$: silicon ${{\boldsymbol{ {\mathcal R} }}}_{{\boldsymbol{D}}}$: silver		${{\boldsymbol{ {\mathcal R} }}}_{{\boldsymbol{C}}}$: air ${{\boldsymbol{ {\mathcal R} }}}_{{\boldsymbol{D}}}$: air		${{\boldsymbol{ {\mathcal R} }}}_{{\boldsymbol{C}}}$: silicon ${{\boldsymbol{ {\mathcal R} }}}_{{\boldsymbol{D}}}$: silicon	
	${{\boldsymbol{\rho }}}_{{{\boldsymbol{P}}}_{{\bf{RS}}}}$	${{\boldsymbol{\rho }}}_{{{\boldsymbol{C}}}_{{\bf{RS}}}}$	${{\boldsymbol{\rho }}}_{{{\boldsymbol{P}}}_{{\bf{RS}}}}$	${{\boldsymbol{\rho }}}_{{{\boldsymbol{C}}}_{{\bf{RS}}}}$	${{\boldsymbol{\rho }}}_{{{\boldsymbol{P}}}_{{\bf{RS}}}}$	${{\boldsymbol{\rho }}}_{{{\boldsymbol{C}}}_{{\bf{RS}}}}$	${{\boldsymbol{\rho }}}_{{{\bf{P}}}_{{\bf{RS}}}}$	${{\boldsymbol{\rho }}}_{{{\boldsymbol{C}}}_{{\bf{RS}}}}$
1.0	0.5620	0.5606	0.6039	0.5576	0.6482	0.6316	0.5868	0.5614
2.0	0.5834	0.5806	0.5357	0.5082	0.6116	0.6020	0.5865	0.5654
3.0	0.5354	0.5310	0.5542	0.5245	0.6335	0.6269	0.5870	0.5759

Both the shape and the duration of the received signal at point S are changed, if silver is abruptly terminated at $x=0$ and both ${ {\mathcal R} }_{C}$ and ${ {\mathcal R} }_{D}$ are occupied by either air or silicon. Table 2 shows ${\rho }_{{P}_{{\rm{RS}}}}$ and ${\rho }_{{C}_{{\rm{RS}}}}$ are ~0.62 for all chosen values of *d*_S_ when ${ {\mathcal R} }_{C}\cup { {\mathcal R} }_{D}$ is occupied by air. The values of ${\rho }_{{P}_{{\rm{RS}}}}$ and ${\rho }_{{C}_{{\rm{RS}}}}$ indicate that the received signal’s shape and duration are close to those of the transmitted signal, which is not surprising because air is nondispersive. The values of ${\rho }_{{P}_{{\rm{RS}}}}$ and ${\rho }_{{C}_{{\rm{RS}}}}$ are lower (~0.57) when ${ {\mathcal R} }_{C}\cup { {\mathcal R} }_{D}$ is occupied by silicon, because silicon is slightly dispersive in the near-infrared regime.

## Concluding Remarks

Motivated by the need for optical interconnections in silicon microelectronics, we numerically investigated the transfer of information by the amplitude modulation of a surface-plasmon-polariton wave guided by a silicon/silver interface, the carrier frequency lying in the near-infrared regime with dissipation of electromagnetic energy in silicon. As the signal pulse propagates guided by the silicon/silver interface, its temporal profile broadens and its amplitude reduces. The broadening is consistent with different spectral components of the signal having different phase speeds, and amplitude reduction occurs due to dissipation of the electromagnetic energy in both silver and silicon. The loss of fidelity, as quantified by the Pearson and concordance correlation coefficients, is not excessive.

The signal is partially reflected and partially transmitted without significant loss of fidelity, when silicon is terminated by air; however, no transmission occurs when silicon is terminated by silver. The fidelity of the transmitted signal in the forward direction rises when both silicon and silver are terminated by air.

Thus, our foundational investigation shows that the information can be transferred over distances on the order of a few tens of micrometers in microelectronic chips by SPP waves. Even if the metal is abruptly terminated, information continues to propagate in the forward direction. Effective strategies to reduce reflections do need to be devised, but even so our results are promising for SPP-wave-based optical interconnections because planar structures for propagating SPP waves for even longer distances have been devised and fabricated^29^.
